# Utilizing machine learning to facilitate the early diagnosis of posterior circulation stroke

**DOI:** 10.1186/s12883-024-03638-8

**Published:** 2024-05-07

**Authors:** Ahmad A. Abujaber, Yahia Imam, Ibrahem Albalkhi, Said Yaseen, Abdulqadir J. Nashwan, Naveed Akhtar

**Affiliations:** 1 Nursing Department, Hamad Medical Corporation (HMC), Doha, Qatar; 2 Neurology Section, Neuroscience Institute, Hamad Medical Corporation (HMC), Doha, Qatar; 3 College of Medicine, Alfaisal University, Riyadh, Saudi Arabia; 4 Department of Neuroradiology, Great Ormond Street Hospital NHS Foundation Trust, Great Ormond St, London, WC1N 3JH UK; 5 School of Medicine, Jordan University of Science and Technology, Irbid, Jordan; 6 Department of Public Health, College of Health Sciences, QU Health, Qatar University, Doha, Qatar; 7 Neuroradiology Department, Neuroscience Institute, Hamad Medical Corporation (HMC), Doha, Qatar

**Keywords:** Posterior circulation syndrome (PCS), Posterior stroke diagnosis, Machine learning, Decision support, Stroke risk factors

## Abstract

**Background:**

Posterior Circulation Syndrome (PCS) presents a diagnostic challenge characterized by its variable and nonspecific symptoms. Timely and accurate diagnosis is crucial for improving patient outcomes. This study aims to enhance the early diagnosis of PCS by employing clinical and demographic data and machine learning. This approach targets a significant research gap in the field of stroke diagnosis and management.

**Methods:**

We collected and analyzed data from a large national Stroke Registry spanning from January 2014 to July 2022. The dataset included 15,859 adult patients admitted with a primary diagnosis of stroke. Five machine learning models were trained: XGBoost, Random Forest, Support Vector Machine, Classification and Regression Trees, and Logistic Regression. Multiple performance metrics, such as accuracy, precision, recall, F1-score, AUC, Matthew’s correlation coefficient, log loss, and Brier score, were utilized to evaluate model performance.

**Results:**

The XGBoost model emerged as the top performer with an AUC of 0.81, accuracy of 0.79, precision of 0.5, recall of 0.62, and F1-score of 0.55. SHAP (SHapley Additive exPlanations) analysis identified key variables associated with PCS, including Body Mass Index, Random Blood Sugar, ataxia, dysarthria, and diastolic blood pressure and body temperature. These variables played a significant role in facilitating the early diagnosis of PCS, emphasizing their diagnostic value.

**Conclusion:**

This study pioneers the use of clinical data and machine learning models to facilitate the early diagnosis of PCS, filling a crucial gap in stroke research. Using simple clinical metrics such as BMI, RBS, ataxia, dysarthria, DBP, and body temperature will help clinicians diagnose PCS early. Despite limitations, such as data biases and regional specificity, our research contributes to advancing PCS understanding, potentially enhancing clinical decision-making and patient outcomes early in the patient’s clinical journey. Further investigations are warranted to elucidate the underlying physiological mechanisms and validate these findings in broader populations and healthcare settings.

## Introduction

Posterior circulation stroke (PCS) constitutes 20% of all ischemic stroke [1] with 70,000–100,000 patients presenting with PCS in the USA annually [2]. PCS is difficult to diagnose owing to the often stuttering, progressive and/or non-lateralizing nature of the symptoms given the vast area of blood supply and non-specific symptomatology [3]. Furthermore, computed tomography (CT), is less reliable in diagnosing PCS [10]. As described by Schneider et al. [4]. , the Timely diagnosis relies upon a careful history and a high clinical index of suspicion (e.g. speed of onset, age, and vascular risk factors).

Research conducted by Mehndiratta and colleagues highlighted hypertension as the predominant risk factor associated with PCS. Interestingly, vertigo emerged as the most frequently observed clinical symptom, closely followed by ataxia [5]. Furthermore, in a comparison with anterior circulation strokes, it was observed that type 2 diabetes mellitus exhibited a stronger association with PCS, particularly in cases of pontine infarctions as opposed to non-pontine subtypes of PCS [6]. Additionally, it’s worth noting that vertebral artery hypoplasia is present in 10% of the general population in China, and it stands as an independent risk factor for PCS, alongside male sex [7].

Qatar, a prosperous peninsula situated on the northeastern border of the Arabian Peninsula, has a native Qatari population comprising only 15% of the total populace [4]. Despite its affluence, the country faces significant public health challenges, including a high prevalence of obesity, diabetes mellitus (DM), and cardiovascular disease [5]. In 2020, Qatar ranked 15th globally for obesity, affecting over 35% of its citizens. Additionally, in 2013, approximately 16% of the population received a diagnosis of diabetes mellitus [6]. Notwithstanding these concerning statistics, Qatar maintains a relatively low stroke incidence rate of 58 cases per 100,000 individuals, significantly lower than the MENA region’s rate of 250 cases per 100,000 people [7, 8]. Moreover, Qatar exhibits a comparatively low rate of stroke-related fatalities [7]. This phenomenon can be ascribed to the distinctive demographic makeup, where the expatriate working-age population constitutes the majority [4, 9]. This heterogeneous demographic and ethnic composition have a significant implication on the stroke characteristics compared to the Caucasian dominant population where most of the published stroke database publications come from [4].

The use of Machine Learning (ML) in medicine, particularly through Explainable Artificial Intelligence (XAI), is crucial for enhancing model performance, building user trust, and supporting decision-making processes, thereby potentially increasing AI’s clinical impact and adoption in healthcare [8]. By developing robust ML models for early and accurate prediction of clinical outcomes across diverse demographics. This advancement enables the customization of treatment plans to meet the unique needs of each patient, thereby potentially saving lives. Giuste et al. developed a robust ML model to predict patient-specific risk of death using features available at the time of diagnosis. Consequently, this fosters a greater acceptance and integration of AI technologies within healthcare frameworks [9].

Various scoring systems and scales have been employed to assess and predict the outcome, prognosis, and severity of PCS. A substantial body of research consistently indicates that PCS tends to carry a less favorable prognosis in comparison to anterior circulation strokes [10]. The National Institutes of Health Stroke Scale (NIHSS) serves as the most widely utilized tool for gauging stroke severity. However, it falls short when assessing PCS due to its inability to capture clinical elements specific to the posterior circulation, such as nystagmus or gait disturbances. This limitation can result in an underestimation of the severity of PCS [11, 12]. To address this gap, several alternative scoring systems have been developed to more accurately evaluate the severity of PCS, such as Adam’s Scale of Posterior Stroke (ASPOS) [12] and the posterior NIHSS [13] Additionally, research has shown that utilizing NIHSS scores 24 h post-stroke proves to be a more precise predictor of functional outcomes within 90 days following thrombectomy, as opposed to relying solely on NIHSS scores upon admission [14].

ML has been heavily utilized in the field of stroke to predict certain stroke outcomes and to help improve personalized medicine [15, 16]. ML-based assessment tools, such as the Posterior Circulation Acute Stroke Prognosis Early CT-Score (pc-ASPECTS), have been developed to enhance outcome prediction for PCS utilizing imaging-based ML. Impressively, pc-ASPECTS demonstrates superior accuracy when evaluated through Receiver Operating Characteristic (ROC) curves, particularly in forecasting outcomes for minor strokes occurring within the initial 36 h after stroke onset. Utilizing a cutoff value of 7, individuals with a pc-ASPECTS score exceeding 7 tend to exhibit more favorable outcomes [17, 18]. Similarly, pcASCO (Posterior Circulation Acute Stroke Prognosis Collateral Score) serves as another imaging-based scoring system designed to predict functional independence at day 90 and the occurrence of malignant cerebellar edema (MCE) in patients with basilar artery occlusion (BAO) stroke upon admission [19]. Furthermore, Tan and colleagues, have identified the Hyperdense Basilar Artery Sign (HDBA) on unenhanced computed tomography scans as a valuable indicator for the early diagnosis of acute PCS and the prediction of a less favorable short-term outcome [20].

While significant strides have been made in predicting stroke outcomes, it is evident that progress in the domain of PCS outcome prediction lags significantly behind that of anterior circulation stroke models. What is more concerning is the limited research dedicated to facilitating the early diagnosis of PCS, primarily due to its challenging presentation. Therefore, this study aims to address this gap by utilizing a diverse range of machine-learning models to boost the physicians’ capacity to diagnose PCS early using patients’ demographic and clinical data without relying on complex imaging data.

## Methods

**Fig. 1:**
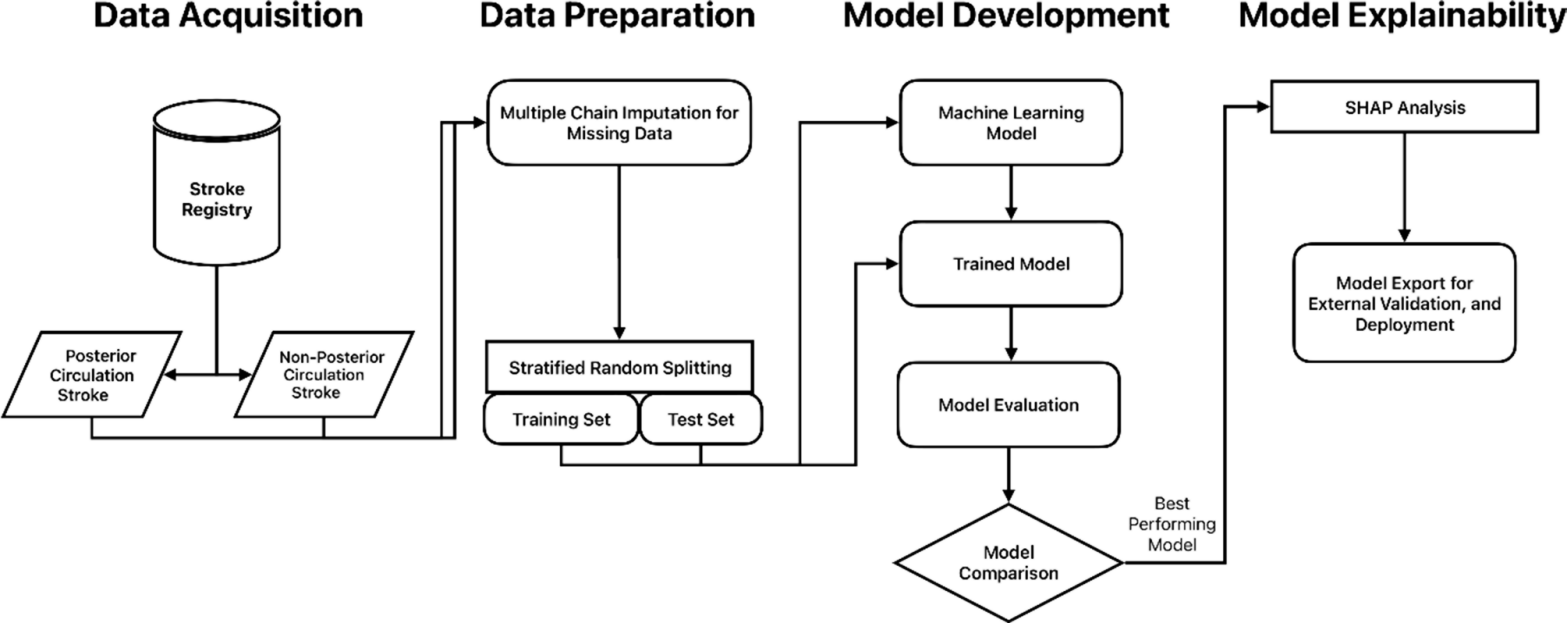
Flow diagram for the proposed prediction system

Our proposed methodology involves a systematic approach to utilize ML techniques for analysing data from a stroke registry. Initially, we gather comprehensive data from the registry, ensuring inclusion of all relevant variables. Following this, we conduct thorough data analysis to uncover correlations among the variables. The dataset is then split for training multiple ML models. These models undergo evaluation, with the best-performing model selected for further analysis and potential deployment. Figure 1 outlines the sequential steps of our ML prediction system.

### Ethical approval

The study obtained authorization from the Institutional Research Board (IRB) at Hamad Medical Corporation in Qatar, identified by reference number MRC-01-22-594.

### Study population

We gathered data from the national Qatar Stroke Registry housed at Hamad General Hospital (HGH), the sole tertiary and referral stroke center in Qatar, covering the period from January 2014 to July 2022. The dataset comprises individuals aged 18 years and above who were admitted to HGH with a primary diagnosis of stroke. Over the course of establishing the stroke registry in Qatar until July 2022, a total of 15,859 patients sought specialized stroke treatment at the hospital. This encompassed patients with diagnoses of ischemic and hemorrhagic strokes, transient ischemic attacks (TIAs), and stroke mimics. However, our study specifically concentrates on patients diagnosed with non-hemorrhagic strokes who have valid Bamford class, while excluding all other conditions.

### Baseline variables

The data collected encompassed a comprehensive array of patient information, spanning demographics, hemodynamic measurements upon admission (including heart rate (HR), blood pressure (BP), and temperature), factors contributing to stroke risk (such as smoking history, pre-existing medical conditions, Body Mass Index (BMI)), neurological symptoms at presentation, and the assessment of stroke severity at admission utilizing the National Institute of Health Stroke Score (NIHSS) [21, 22]. We utilized the CDC’s 5-class definition for adult overweight and obesity [23] to categorize Body Mass Index (BMI) groups.

Regarding ethnicity, patients were categorized into five distinct groups based on their reported nationality: Qatari, Middle East and North Africa (MENA) region, South Asia region, South East Asia region (defined in accordance with the United Nations geo-scheme), and all other nationalities were grouped into an “other” category [24, 25]. Notably, the specific categorization for Qatari patients was employed to enable meaningful comparisons, recognizing the unique demographic composition of the country where a significant portion of the population consists of expatriates [26, 27]. This classification methodology has been consistently applied in previous stroke research in Qatar [15, 24, 28].

All relevant risk factors, such as pre-existing medical conditions and smoking history, were meticulously recorded during the patient’s hospitalization and cross-validated by stroke registry personnel through electronic medical records. A total of 29 variables, as outlined in Table  1, were utilized in predicting PCS.

### Outcome measure

Our primary focus for outcome is diagnosing POCI (PCS) based on the Bamford classification of cerebral stroke. Bamford classification, also known as, Oxford Community Stroke Project Classification, classifies the cerebral infarction stroke into four categories prognostically and etiologically as follows; (a) Total Anterior Circulatory Infarct (TACI), (b) Lacunar Anterior Circulatory Infarct (LACI), (c) Partial Anterior Circulatory Infarct (PACI), and (d) Posterior Circulatory Infarct (POCI) [29–31]. In this study’s outcome variable, PCS cases were coded as ‘1’, while all other classes were assigned a ‘0’ code.

### Inclusion/exclusion criteria

**Fig. 2:**
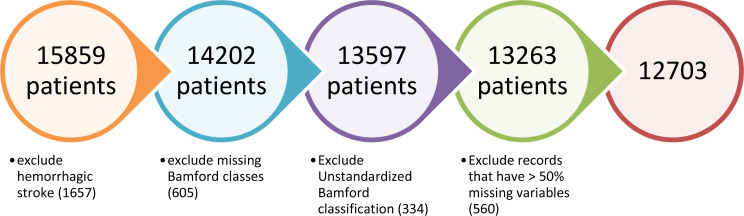
Inclusion and exclusion process

This study encompassed all adult patients aged 18 years or older who received a diagnosis of stroke. Out of the initial cohort of 15,859 patients, 1,657 who were diagnosed with haemorrhagic stroke were excluded. Records with missing or unstandardized outcome variables were also eliminated. Additionally, any records that lacked more than 50% of the included variables were excluded, resulting in a final dataset of 12,703 records eligible for the study, as depicted in Fig. 2.

### Handling missing data and class imbalance

In instances where data value was missing, we adopted the Multiple Imputation using Chained Equations (MICE) technique to generate data imputations [32]. Within the dataset, it was observed that the Random Blood Sugar (RBS) had the highest rate of missing values at 3% and followed by Heart Rate (HR) at 0.3%. The cohort presented a PCS rate of 20.7%, leading to a concern regarding class imbalance. To address this issue, To address this issue, we integrated class weighting to counteract the imbalance [33, 34]. Specifically, we assigned class weights inversely proportional to class frequencies, granting greater weight to the minority class (patients who have PCS) to enhance their impact during the training process.

### Model training and evaluation

The dataset was divided into two subsets: a training set comprising 80% of the data and a validation set containing 20%, employing a stratified random sampling method. Our models were built using the training dataset, and their performance was assessed using the validation dataset. In order to optimize model performance and efficiency, feature scaling with data normalization was conducted prior to the training of ML models. We trained a total of five machine learning models, including XGB, weight-adjusted RF, SVM, CART, and LR coupled with random under-sampling of the majority class.

We employed a range of classification metrics to evaluate the effectiveness of the models. These metrics included accuracy, precision, specificity, recall, F1-score, area under the receiver operating characteristic curve (AUC), Matthew’s correlation coefficient (MCC), log loss, and Brier score [35–39]. These metrics offer insights into the model’s ability to accurately classify both positive instances (patients with PCS) and negative instances (patients with non-PCS), considering the class imbalance. The model with the highest F1-score will be selected as the primary model for subsequent external and temporal validation.

### Model explainability

To gain better insight into the decision-making of ML models, we utilized SHAP (SHapley Additive exPlanations). SHAP is a robust toolkit employed to elucidate the classification made by ML models [40]. It works by producing “variable importance” scores at the individual level for features, known as SHAP values. These values quantify how much each feature contributes to a specific classification result.

**Table 1:** Statistical characteristics of the collected stroke dataset

Variable	Feature	Bamford (no PCS)	Bamford (PCS)	Total
Age	< mean (53.7)	5313	1261	6574
	≥mean (53.7)	4750	1379	6129
	Mean ± SD (53.7 ± 14.35)-IQR 20			
Sex	1: Male	7231	2072	9303
	2: Female	2832	568	3400
Ethnicity	1: Qatari	2089	486	2575
	2: MENA	2411	573	2984
	3: South Asian	4208	1242	5450
	4: South-East Asian	819	222	1041
	5: Other	536	117	653
NIHSS at admission	< mean (3.42)	7191	1873	9064
	≥mean (3.42)	2872	767	3639
	Mean ± SD (3.42 ± 5.3)-IQR 4			
Body Mass Index (BMI)	1: Underweight	57	1	58
	2: Normal weight	1365	100	1465
	3: Overweight	2035	164	2199
	4: Obese	1206	95	1301
	5: Extremely Obese	783	52	835
Diabetes Mellitus (DM)	0: No	5253	1123	6376
	1: Yes	4810	1517	6327
Hypertension (HTN)	0: No	3930	721	4651
	1:Yes	6133	1919	8052
Dyslipidemia	0: No	5868	1451	7319
	1: Yes	4195	1189	5384
Prior stroke	0: No	8765	2352	11,117
	1: Yes	1298	288	1586
Atrial Fibrillation (AF)	0: No	9581	2525	12,106
	1: Yes	482	115	597
Coronary Artery Disease (CAD)	0: No	8904	2314	11,218
	1: Yes	1159	326	1485
Congestive Heart Failure (CHF)	0: No	10,012	2625	12,637
	1: Yes	51	15	66
Renal Failure	0: No	9863	2575	12,438
	1: Yes	200	65	265
Tobacco use	0: No	8055	2059	10,114
	1: Yes	2008	581	2589
Random Blood Sugar (RBS) at admission	< mean (8.7)	6748	1404	8152
	≥ mean (8.7)	3046	1130	4176
	Mean ± SD (8.7 ± 4.5)-IQR 4.5			
Systolic Blood Pressure (SBP) at admission	< mean (150.5)	5762	1259	7021
	≥ mean (150.5)	4296	1377	5673
	Mean ± SD (150.5 ± 29)-IQR 38			
Diastolic Blood Pressure (DBP) at admission	< mean (88)	5437	1308	6745
	≥ mean (99)	4619	1326	5945
	Mean ± SD (88 ± 18)-IQR 22			
Heart Rate (HR) at admission	< mean (82)	5486	1480	6966
	≥ mean (82)	4557	1137	5694
	Mean ± SD (82 ± 14.9)-IQR 18			
Body Temperature (°C)	< mean (36.7)	5156	1441	6597
	≥ mean (36.7)	4892	1177	6069
	Mean ± SD (36.7 ± 0.33)-IQR 0.4			
Aphasia	0: No	9459	2589	12,048
	1: Yes	604	51	655
Neglect	0: No	9964	2622	12,586
	1: Yes	99	18	117
Gaze Deviation	0: No	9932	2616	12,548
	1: Yes	131	24	155
Hemianopia	0: No	9893	2568	12,461
	1: Yes	170	72	242
Ataxia	0: No	9681	2086	11,767
	1: Yes	382	554	936
Diplopia	0: No	9725	2353	12,078
	1: Yes	338	287	625
Dysphagia	0: No	10,004	2595	12,599
	1: Yes	59	45	104
Dysarthria	0: No	7516	2136	9652
	1: Yes	2547	504	3051
Loss of Consciousness	0: No	9593	2518	12,111
	1: Yes	470	122	592
Seizure	0: No	9876	2615	12,491
	1: Yes	187	25	212
Bamford Classification	0: Non-PCS 1: PCS	10,063	2640	12,703

**Table 2:** Models performance evaluation metrics

Model	Accuracy	Precision	Specificity	Recall	F1-Score	AUC	MCC	Log Loss	Brier Score
XGB Classifier	0.79	0.5	0.83	0.62	0.55	0.81	0.41	0.45	0.15
Random Forest Classifier (RF)	0.83	0.72	0.97	0.27	0.39	0.85	0.36	0.37	0.12
Support Vector Machine (SVM)	0.72	0.41	0.71	0.77	0.54	0.82	0.40	0.40	0.13
Decision Tree Classifier (CART)	0.77	0.45	0.85	0.47	0.46	0.66	0.31	8.24	0.23
Logistic Regression (LR)	0.72	0.41	0.71	0.77	0.53	0.82	0.39	0.53	0.18

## Results

The cohort includes 12,703 patients. As in Tables 1 and 73% of the patients are males. The mean age of the cohort is 53.7 ± 14 years. Approximately, 21% of the patients were diagnosed with PCS consistent with the global trend. Around 44% of the cohort is from MENA region and 43% is from South Asian ethnicity.

### Model evaluation

**Fig. 3:**
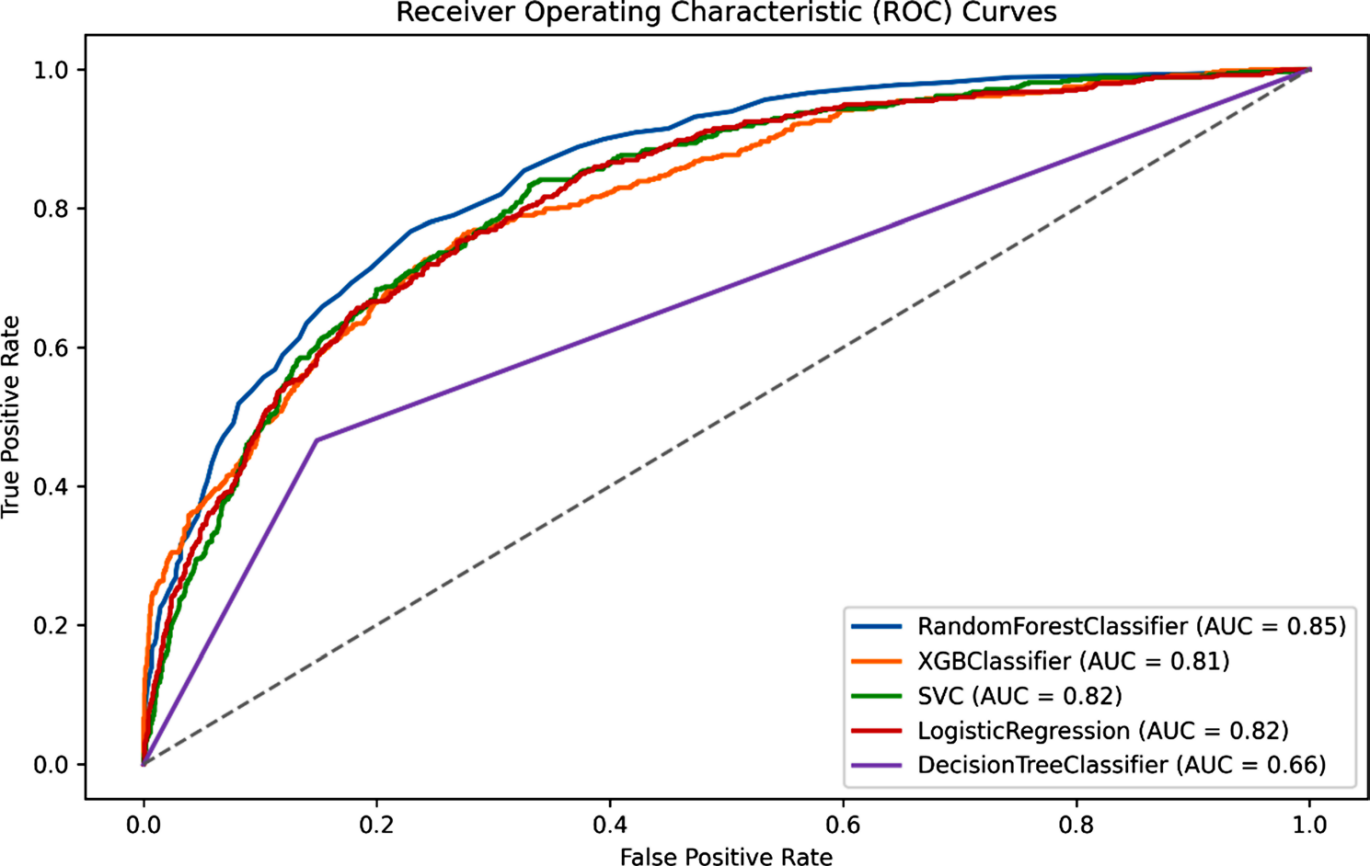
Area Under the Receiving Operating Curve (AUC-ROC) of the trained models

As indicated in Table 2; Fig. 3, the models exhibit varying levels of performance, with the XGBoost (XGB) model emerging as the top performer. Despite the moderate F1 score and precision, XGB achieved notable results, boasting an AUC of 0.81, an accuracy of 0.79, a precision score of 0.5, a recall rate of 0.62, and an F1-score of 0.55. The Random Forest (RF) model also demonstrated competitive performance, with an AUC of 0.83, though it recorded the lowest F1 score of 0.39, indicating relatively weaker overall classification capabilities. Conversely, Logistic Regression (LR) excelled in terms of AUC, recall, Brier score, and F1 score but exhibited lower precision, specificity, and Matthew’s Correlation Coefficient (MCC). Therefore, we have chosen XGB as the primary model for subsequent SHAP analysis, external validation, and temporal validation in diagnosing PCS.

### SHAP analysis

**Fig. 4:**
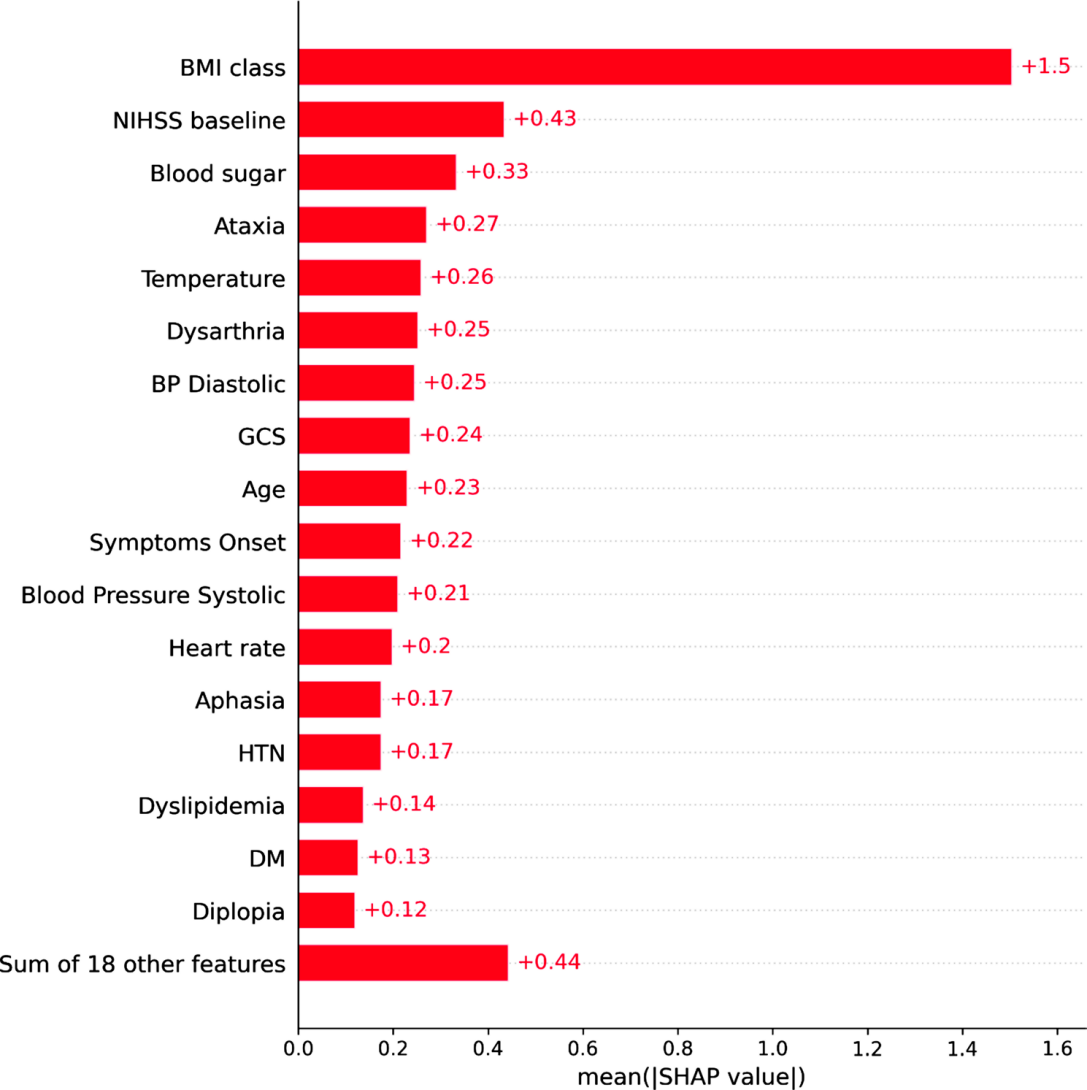
SHAP mean values

**Fig. 5:**
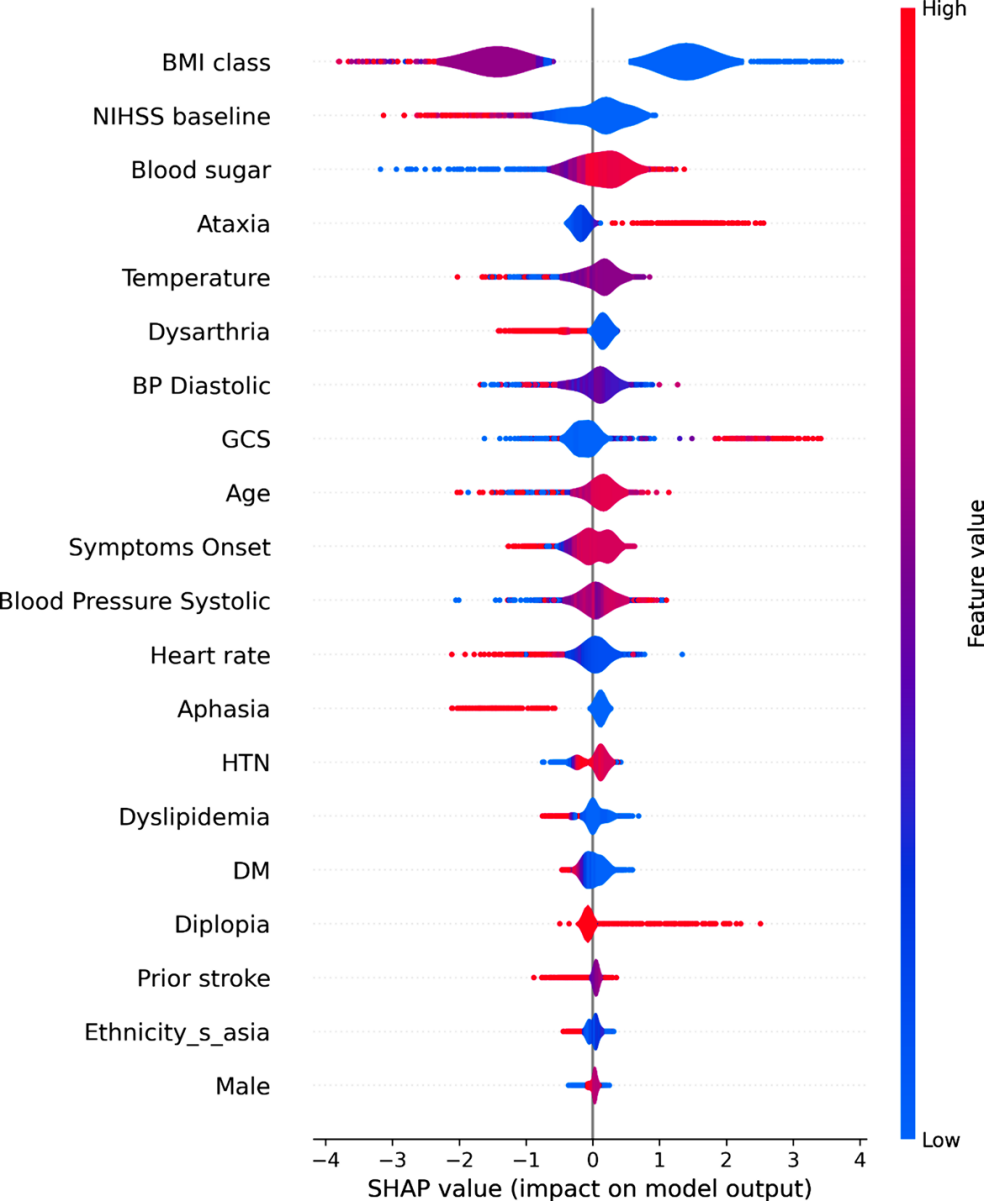
SHAP violin plot- variables impact on model’s output

The SHAP analysis yielded invaluable and pivotal insights for identifying PCS. In this context, the crucial factors, ranked by importance, are BMI, stroke severity upon presentation (measured by NIHSS), Random Blood Sugar (RBS), ataxia, dysarthria, Diastolic Blood Pressure (DBP), and body temperature at admission. Figures 4 and 5 illustrate SHAP analysis results.

## Discussion

We evaluated the effectiveness of five ML models and harnessed SHAP analysis to determine the pivotal factors linked to PCS. The aim is to facilitate early identification of PCS which is challenging to diagnose through ML models utilizing simple clinical data without neuroimaging. The paucity of existing literature addressing utilization of simple clinical data to classify PCS diagnosis underlines the distinctiveness and originality of our study

SHAP analysis reaffirmed the significant influence of BMI on the model’s classification performance. Typically, obesity is well-recognized as a leading risk factor for stroke and is linked with unfavorable prognoses [41, 42]. A study conducted in Qatar in 2009 similarly identified obesity as one of the major risks associated with PCS, finding that patients with a BMI > 30 faced a heightened risk of PCS compared to those with lower BMIs [43]. Likewise, our study revealed that patients with a BMI ≥ 25 are four times more likely to be at a higher risk of PCS compared to those with a BMI < 25. Remarkably, 22.7% of patients presenting with PCS had a BMI greater than 25%, in contrast to only 6.6% with a BMI below 25%. This difference was statistically significant with a p-value less than 0.05. However, it is important to acknowledge recent reviews that have questioned the robustness of BMI as an indicator of obesity. These critiques cite concerns of potential inaccuracies in body weight estimation, insufficient adjustments for comorbidities, non-linear BMI-outcome relationships, limited follow-up durations, and potential selection bias arising from the retrospective nature of much of the earlier literature [44, 45]

The National Institute of Health Stroke Scale (NIHSS) is a well-established tool for assessing stroke severity and predicting stroke outcomes [15, 16]. In our study, we observed that PCS tends to be associated with a slightly lower NIHSS scores upon admission when compared to others, as depicted in Fig. 5, with PCS averaging 3.25 on the NIHSS score, while other stroke types averaged 3.43, however this was not statistically significance. Similarly, Wen-Dan et al. and Imam et al. found that PCS to be associated with lower NIHSS at admission [46, 47]. This is probably due to its inherent bias of design towards anterior circulation resulting in higher scores assigned to anterior circulation strokes rather than PCS [48]

The third variable that exhibited a significant association with PCS and contributed to the model’s output is the Random Blood Sugar (RBS) level. The anterior and posterior circulations in the body differ anatomically and functionally from the posterior circulation. Anatomically, it is common to observe hypoplasia (underdevelopment) or aplasia (absence) in the vertebral artery. Frequently, one of these two arteries is either underdeveloped or terminates prematurely at the posterior inferior cerebral artery. These thinner, underdeveloped arteries are more susceptible to thrombotic occlusion, especially in the context of poor glycemic control. Functionally, the posterior circulation exhibits a less effective self-regulating mechanism for maintaining stable blood flow [49], and it also receives reduced influence from the sympathetic nervous system [50]. This makes the posterior circulation more susceptible to damage in diabetics. Diabetes also causes different structural changes in the anterior and posterior circulations, influenced by glycated hemoglobin [51], increasing the risk of stroke in the posterior circulation due to chronic high blood sugar [47]. This study identified a positive correlation between RBS levels and PCS. In our secondary analysis, we observed that the average RBS measured at admission for patients with PCS was significantly higher than that for those with non-PCS, with values of 10 mmol/l compared to 8.5 mmol/l, p-value < 0.05

Symptoms presented during initial patient assessment play a crucial role in aiding physicians in the diagnostic process. Our study revealed that ataxia emerges as a key indicator for the early diagnosis of PCS. Remarkably, 59.2% of PCS patients manifested ataxia, in contrast to 41% of patients in other diagnostic categories. Importantly, the odds of experiencing ataxia in cases of PCS were six times higher than in non-PCS cases, with a p-value < 0.05. This finding aligns with previous research, where the presence of ataxia has consistently been associated with PCS, regardless of the specific location of the lesion [5, 52]

Similarly, our analysis revealed that dysarthria was more strongly associated with non-PCS categories as opposed to PCS. Specifically, only 16.5% of patients diagnosed with PCS exhibited dysarthria, while a significant majority, accounting for 83.5%, was observed in non-PCS cases, p-value < 0.05. Interestingly, Wen-Dan et al. did not identify a significant distinction between PCS and anterior circulation strokes in terms of the presentation of dysarthria [46]. Dysarthria is a nonspecific symptom that occurs in stroke [53] and in many non-vascular brain disease such Parkinsons Disease [54], Traumatic brain injury [53] and Motor neuron disease [55] as well as stroke mimics [56]. It’s worth noting that our findings may be attributed to the focus of our study on PCS versus all other non-hemorrhagic stroke types rather than anterior stroke alone as in Wen-Dan et al.’s study. And given lacunar syndromes that are commonly associated with dysarthria such as clumsy-hand dysarthria are typically classified as lacunar stroke rather than POCI this maybe an additional reason for its prevalence in non-PCS stroke

Diastolic Blood Pressure (DBP) was found to be strongly associated with PCS, with patients diagnosed with PCS typically presenting higher mean DBP levels than those in other diagnostic categories. For the entire cohort, we observed a mean DBP of 88 ± 18 mmHg. Notably, patients with PCS exhibited a mean DBP of 89 mmHg, compared to 87.7 mmHg in patients from other diagnostic categories, p-value < 0.05. Age and lacunar etiology are both associated with systolic hypertension whereas in this selective relatively young population where presenting phenotypical lacunar strokes are grouped as non-PCS, we found an association with an elevated DBP [57]. Similarly, a study conducted in South London reported a noteworthy association between DBP readings, both before and after the occurrence of PCS, in comparison to anterior circulation stroke cases [58]

Past literature has examined the relationship between body temperature and acute stroke, with findings suggesting that body temperature can serve as an independent predictor of stroke outcomes, where higher temperatures are associated with poorer outcomes [59, 60]. However, there has been a very limited prior research reporting differences in body temperature readings upon admission between PCS and other non-hemorrhagic stroke categories such as in Karaszewski et al. [61, 62] and Kim eta al [63]. , . This gap in research could stem from healthcare providers primarily concentrating on distinguishing between normal and abnormal hemodynamic values, rather than identifying subtle variations within the normal range. These minor changes in hemodynamic readings, although within normal limits, may have potential correlations with various clinical conditions. In our study, the mean temperature for the entire cohort stood at 36.7 ± 0.33 °C. Our secondary analysis revealed a statistically significant difference in mean body temperature between patients with PCS and those with other stroke types. Specifically, the mean body temperature for the PCS cohort was 36.6, compared to 36.72 for the other cohort, with a p-value < 0.05. The remaining variables, although important, were found to have lesser influence on the model’s classification performance

## Strength and limitations

The strength of this study lies in its creation of a forward-thinking, comprehensive, and diverse dataset, which is distinguished by its formation as a nationwide database. This approach effectively surpasses the constraints typically associated with traditional hospital-centric registries. This study employed a national stroke registry, prospectively capturing data from all patients receiving specialized stroke care across the country. However, our research encounters limitations stemming from the retrospective method of data extraction and the presence of incomplete data records. Furthermore, the use of registries can lead to potential challenges in interpretation, documentation, and coding accuracy

Firstly, our study relies on retrospective integration of clinical data, which may be subject to inherent biases and limitations associated with data collection methods. The accuracy and completeness of the electronic medical records could impact the quality of the data, potentially introducing information bias or missing relevant variables

Secondly, the study’s dataset is drawn from a specific healthcare setting in Qatar, and its generalizability to broader populations or healthcare systems may be limited. Regional variations in stroke demographics, risk factors, and healthcare practices could influence the external validity of our findings. Moreover, our study focuses on the utilization of clinical data and ML models to classify PCS. While this approach yields promising results, it does not necessarily take into consideration the underlying physiological mechanisms or causative factors driving the observed associations. Further research may be needed to elucidate the biological underpinnings of these relationships

Additionally, our study does not account for potential confounding factors that could influence the associations between the variables and PCS. Factors such as medication usage, or lifestyle factors were not included in our analysis but may play a role. Lack of imaging information may also present a significant limitation to the potential of the ML model capacity to achieve better performance

Finally, while ML models offer predictive capabilities, their interpretability may be limited, and the model’s decisions may not always align with clinical reasoning. Ensuring that the predictions generated by these models are clinically relevant and actionable is an ongoing challenge in the field of predictive analytics. Considering these limitations, our study serves as a foundational exploration of PCS classification using clinical data and ML but underscores the need for further research, prospective studies, and consideration of broader contexts and variables in future investigations

## Conclusions

In conclusion, this study represents an original effort to help classify and differentiate PCS upon admission to the hospital using clinical data and ML models. The significance of this study lies in exploring a potential supportive role of ML in helping clinicians with the challenging diagnosis of PCS, based on simple clinical data without neuroimaging. Our analysis of five ML models, accompanied by SHAP analysis, has contributed to a deeper understanding of how these models can aid healthcare providers in the early detection of PCS, addressing a critical gap in stroke research

Several key findings have emerged from our investigation. BMI, stroke severity (NIHSS), RBS, ataxia, dysarthria, DBP and body temperature have been identified as important factors associated with PCS.

## Data Availability

The datasets generated and/or analyzed during the current study are available from the corresponding author on reasonable request and subject to appropriate ethical approvals.
